# Case of Poisoning with Arsenious Acid

**Published:** 1865-11

**Authors:** R. C. Hamill


ARTICLE XXXVIII.

### CASE OF POISONING WITH ARSENIOUS ACID.

Reported to the Chicago Medical Society, by R. C. HAMILL, M.D.

Published by request of the Society.

June 20th, 1865.—Mrs. H., an Irishwoman, aged 25 years, procured at a drug store, on State street of this city, about one ounce of arsenious acid, mixed it in a teacup nearly full of cold water, stirring it well with her finger, then drank, immediately, the whole of it, with the exception of what remained in the bottom of the cup. She did not wait for it to settle, and, according to her account, there was not more than one or two drachms left in the vessel, so that she must have swallowed six or seven drachms. She coolly informed a neighbor that she had taken poison, left a message for her husband, and resigned herself to her bed to await the issue. I was summoned to her bedside, about 1 o'clock P.M., a half-hour after she had swallowed the poisonous draught. She complained of burning pain in the stomach, and pain in the head; pulse 50 and soft. I sent her husband to the nearest drug store for the hydrated per-oxide of iron and a bottle of lime-water. A mustard emetic was given immediately, which emptied the stomach in less than five minutes of more than a pint of semi-fluid ingesta and mucus. She had not eaten anything for more than 24 hours. Her husband returned, bringing only the lime-water—℥iv were given immediately. The hydrated per-oxide of iron was eventually procured, at Dr. Mahla's drug store, on State street, and about 50 minutes after taking the arsenic, the first portion was given. (The preparation of iron was fresh, and *moist* or *pulpy.*) One tablespoonful was administered every five minutes, until four or five portions were taken, and the residue of five ounces was given, one tablespoonful every ten minutes.

At 4 o'clock P.M., she was stupid, extremities cold and numb, pulse small and thready, increased pain and burning in the cardiac portion of the stomach, pain and tenderness over the entire abdomen, and muscular soreness of the legs. Her stomach bore the iron well until she had taken 5 oz., when she refused to take any more, complaining of its weight in the stomach. Tablespoonful doses of lime-water were given every 30 minutes, until 8 o'clock, when I again saw her. She had had three spasms during the last hour, the first one very severe, the second and third consecutively lighter. I remaned a half-hour, during which time she vomited twice, throwing up a little bloody mucus each time. She complained of constriction of the œsophagus, and had a slight spasm, confined to the muscles of the thighs and legs. I ordered infusion of slippery elm bark to be freely drank, and the lime-water to be continued through the night at intervals of one hour—her husband to report to me at 11 P.M., which he did, to the purport that she had not vomited after the first draught of the elm infusion; no more spasms; had slept for almost one hour.

21st.—Tongue and fauces very red; gums swollen and sore; free secretion of saliva, as if salivated; complained of cutting pains through the bowels; pulse fuller and regular. Had slept three or four hours during the night. No appetite—drank a half cup of black tea for breakfast. Ordered barley-water, with which, and the elm infusion, she was to relieve her thirst, which was urgent, during the day. She took no more medicine, except a solution of sulph. magnesia with morphine, on the 22d, to move the bowels. On the 26th, she was able to attend to her domestic duties.

